# The effect of loving-kindness meditation on employees’ mindfulness, affect, altruism and knowledge hiding

**DOI:** 10.1186/s40359-022-00846-0

**Published:** 2022-05-29

**Authors:** Chao Liu, Hao Chen, Yu-Chao Liang, Szu-Erh Hsu, Ding-Hau Huang, Chia-Yi Liu, Wen-Ko Chiou

**Affiliations:** 1 School of Journalism and Communication, Hua Qiao University, Xiamen, 361021 China; 2 School of Film Television and Communication, Xiamen University of Technology, Xiamen, 361021 China; 3 Business Analytics Research Center, Chang Gung University, Taoyuan, 33302 Taiwan; 4 Department of Industrial Design, Chang Gung University, Taoyuan, 33302 Taiwan; 5 Institute of Creative Design and Management, National Taipei University of Business, Taoyüan, 22058 Taiwan; 6 Department of Psychiatry, Chang Gung Memorial Hospital, Taipei, 10507 Taiwan; 7 Department of Industrial Engineering and Management, Ming Chi University of Technology, New Taipei, 24301 Taiwan

**Keywords:** Loving-kindness meditation, Mindfulness, Affect, Altruism, Knowledge hiding

## Abstract

**Background:**

This study investigated the effects of the loving-kindness meditation (LKM) on employees’ mindfulness, affect, altruism and knowledge hiding.

**Methods:**

In total, 100 employees were recruited from a knowledge-based enterprise in China and randomly divided into the LKM training group (n = 50) and the control group (n = 50). The LKM training group underwent LKM training for 8 weeks, while the control group did not. Seven main variables (mindfulness, altruism positive affect, negative affect, playing dumb, rationalized hiding, and evasive hiding) were measured both before (pre-test) and after (post-test) the LKM training intervention.

**Results:**

The LKM intervention significantly increased participants’ altruism, and significantly reduced negative affect, playing dumb and evasive hiding, but did not significantly improve mindfulness, positive affect, and rationalized hiding.

**Conclusions:**

LKM significantly improved employees’ altruism, and significantly reduce their negative affect, but did not significantly improve their mindfulness and positive affect. For knowledge hiding, LKM significantly reduced playing dumb and evasive hiding, but had no significant effect on rationalized hiding. These results further elucidate the psychological effects of LKM and suggest the possibility of reducing knowledge hiding in the workplace.

**Trial registration:**

ChiCTR2200057460. Registered in Chinese Clinical Trial Registry (ChiCTR), 13 March 2022—Retrospectively registered.

## Introduction

At present, with the intensification of competition among enterprises, the competition between enterprises is more dependent on the knowledge mastered by their employees. Knowledge plays an important role in competition among enterprises. Knowledge is the most important resource for an enterprise, which can create inestimable profits and value for the enterprise [1]. However, the knowledge of enterprise innovation is mastered by individual employees. Enterprises cannot force employees to share their knowledge with other members, which makes the organization face an irreconcilable contradiction between “encouraging disclosure” and “resisting concealment”, which eventually leads to the widespread phenomenon of knowledge hiding in enterprises [2]. Although enterprises have taken various measures to encourage employees to share knowledge, such as incentives (material and non-material incentives), creating a fair organizational atmosphere, improving employees’ psychological identification with the organization and establishing a knowledge sharing atmosphere, little effect has been achieved [3]. According to the definition of Connelly et al., knowledge hiding refers to the behavior in which an organization’s members deliberately conceal knowledge when faced with a request for knowledge from colleagues for some purpose. This behavior is mainly manifested in three modes: playing dumb, rationalized hiding and evasive hiding [4]. Studies have shown that knowledge hiding can hinder the exchange and flow of knowledge by building “information barriers”, and has a negative impact on creativity, innovation behavior and interpersonal trust at all levels of the organization [5]. In view of the importance of knowledge resource management, the universality of the knowledge hiding phenomenon and the harm of knowledge hiding behavior, in recent years, academic circles have explored the question of why employees choose knowledge hiding. It has been found that the emotional state of employees is an important factor of knowledge hiding [6].

This study explored the influencing mechanism of loving-kindness meditation (LKM) on employees' mindfulness, affect, altruism and knowledge hiding, and provided a new theoretical perspective for further understanding the internal mechanism of LKM’s influence on employees' knowledge hiding. In practice, it can enhance the effectiveness of acquiring knowledge, prevent knowledge loss, better allocate knowledge resources and improve the core competitiveness of the organization. The biggest difference between this study and previous studies lies in the introduction of LKM, mindfulness, positive affect from positive psychology and altruism from social psychology into organizational psychology to study knowledge hiding behavior in workplace employees, and the use of an experimental design to verify the causal relationships among relevant variables to fill the research gaps in related fields.

### The broaden-and-build theory of positive affect

Barbara Fredrickson first proposed and developed the broaden-and-build theory of positive affect [7]. With the rise of positive psychology and positive organizational scholarship, the broaden-and-build theory of positive affect has been paid more and more attention in theoretical and practical circles, and has become the basic theory explaining how positive affect bring positive effects to individuals and organizations [8].

Affect is a feeling or emotional state that a person experiences subjectively [7]. Negative affect (such as anger, fear, disgust, guilt, etc.) have been studied extensively by psychologists before the development of the broaden-and-build theory of positive affect [9]. Basically, each of the basic types of negative affect corresponds to a specific action tendency, such as anger inducing attack and fear leading to flight. In life-threatening situations, negative affect narrows the momentary thought–action repertoire of individuals and enhances their ability to act quickly and firmly, thus having evolutionary significance. For example, in a dangerous situation, a particular tendency to act aroused by negative affect saved the lives of our ancestors, increasing an individual’s chances of survival [10]. Fredrickson believes that although specific behaviors can explain most of the form and function mechanism of negative affect, this approach is not suitable for positive affect, because positive affect does not seem to stimulate a response to life-threatening conditions and does not usually point to a particular behavior. Therefore, positive affect must be different from negative affect in terms of adaptive significance [11]. Therefore, Fredrickson proposed the broaden-and-build theory of positive affect. In general, the broaden-and-build theory of positive affect believes that positive affect can expand the instantaneous thinking and action range of individuals, and then construct lasting personal resources (intellectual resources, physical resources, psychological resources and social resources), thus bringing long-term adaptive benefits for individuals. There are two core assumptions of broaden-and-build theory [12], namely the broaden hypothesis and the build hypothesis. The broaden hypothesis holds that, compared with negative affect and neutral states, positive affect can expand individuals’ instantaneous mind–action range (positive affect can expand individuals’ attention, cognition and action range) and can make individuals' thinking patterns become unusual, flexible, tolerant and empathetic [12]. According to the build hypothesis, the expanded instantaneous thought–action range can help individuals build lasting social resources, not only to consolidate existing social connections and establish new social connections, etc., but also to construct more mindfulness and altruistic behaviors [12].

### Loving-kindness meditation

As a traditional meditation method with an important position in Buddhism and great application potential in practice, compassionate meditation has attracted more and more attention from Western psychologists such as Hofmann, Grossman and Hinton [13]. LKM and mindfulness have many similarities. Kabat Zinn believed that they can both be regarded as a method of attention and emotion regulation [14].

LKM refers to a special meditation practice that cultivates compassion. This kind of compassion is a type of unconditional and undifferentiated goodwill toward all sentient beings, that is, this kind of goodwill is not affected by relationships or interests [15]. LKM emphasizes the cultivation of “compassion”, “appreciative joy” and “acceptance”, which are noble attitudes toward all sentient beings [16]. Compassion is defined as compassion for the unfortunate, appreciative joy is joy at the happiness or success of others, and acceptance is equanimity or calm. In life and work, when the mood is low, LKM can be practiced to adjust the immediate mood, and long-term practice of LKM is conducive to long-term mood regulation [17].

Few studies have explored the effects of LKM on mindfulness and positive affect. Sorensen’s research showed that LKM could improve college students’ mindfulness [18]. Kabat Zinn defined mindfulness as “a state of consciousness produced by directing attention to a goal in the present moment, manifested as a nonjudgmental treatment of various experiences or unfolding in the present moment”. Currently, the concept of mindfulness includes two elements of positive psychology: acceptance and non-judgment [14]. As a result, mindfulness-based cognitive behavioral therapy has been developed. Previous studies have shown that there was a negative correlation between mindfulness and knowledge hiding among employees of German companies [19]. Although there is evidence that mindfulness and knowledge hiding are negatively correlated, studies exploring the mechanisms behind this relationship are limited. Positive affect and altruism may help explain the relationship between mindfulness and knowledge hiding, a topic that deserves further research [20]. Positive affect falls under the category of pleasure, reflecting the hedonic tones of subjective experience, so the types of positive affect are those that are pleasurable and cause us to engage in behaviors that we like. "Happiness" is the most frequently used word to describe positive experiences in people's daily communication [21]. The emotional experience contained in positive affect can be more accurately interpreted and conveyed by words such as joy, inspiration, gratitude and love [22]. Previous research has explored the precursors of positive affect and found that mindfulness can boost an individual’s positive affect. Mindfulness training can reduce knowledge hiding by enhancing positive affect. Mothers with higher levels of mindfulness usually display stronger activation of a positive emotional environment [23]. Mindfulness improved the positive affect of middle-aged Americans [24], introduced flexibility by enhancing the production of cognitive assessments [25], enhanced the positive affect of patients with social anxiety [26] and improved the positive affect of Chinese college students [27]. Mindfulness was negatively correlated with emotional exhaustion in call center workers in the Philippines [28], anxiety in English speech class in Thai college students [29] and negative emotions regarding organizational change in Thai banking employees [30]. The literature has also revealed the impact of positive affect on knowledge hiding. The results showed that positive affect was negatively correlated with knowledge hiding in Indonesian enterprises [31]. Taken together, these previous findings strongly suggest that people with high levels of mindfulness experience higher levels of positive affect, thereby reducing their knowledge hiding.

In addition to positive affect as a factor that may help explain the relationship between mindfulness and knowledge hiding, altruism may play a mediating role in mindfulness and knowledge hiding. Altruism is the behavior in which individuals help others out of their own free will regardless of external interests [32]. Sometimes, people are willing to help others for nothing. Altruistic behavior occurs even when someone does not know the person he/she is helping, or when even though his/her altruistic act offers no foreseeable benefit, he/she chooses to help [33]. Previous research has explored the precursors of altruism and found that mindfulness can boost personal altruism. Mindfulness training can reduce knowledge hiding by enhancing altruism. Mindfulness improved the altruistic behavior of Americans [34], improved the altruistic behavior of Swedes [35], enhanced an enduring experience of selflessness and service to others in Australians [36], and enhanced the universal human capacity for altruistic experience, love and compassion [37]. Mindfulness was positively correlated with the altruistic behavior of German business leaders [37]. Mindfulness also improved the appearance of intrinsic altruism in Argentine children [38]. Some literature has also revealed the influence of altruism on knowledge hiding. Research showed that the altruism of employees in Croatia’s knowledge-intensive enterprises was positively correlated with knowledge sharing [39], and the altruism of employees in Taiwan's high-tech industries was positively correlated with knowledge sharing [40]. The altruism of high school students in Hong Kong had a direct and significant impact on online knowledge sharing [41], while the altruism of Spanish workers was positively correlated with knowledge sharing [42]. In a professional virtual community of Taiwanese teachers, altruism was positively correlated with knowledge sharing [43], while Indonesian managers’ altruism was negatively correlated with employees' knowledge hiding [31]. Taken together, these previous findings strongly suggest that people with high levels of mindfulness experience higher levels of altruism, thereby reducing their knowledge hiding.

## Research gap and hypotheses

Based on the above literature theories and research findings, this study proposes the following hypotheses:

### Hypothesis 1 (H1)

The LKM intervention can significantly improve the level of mindfulness of the subjects.

### Hypothesis 2 (H2)

The LKM intervention can significantly improve the level of altruism of the subjects.

### Hypothesis 3 (H3)

The LKM intervention can significantly improve the level of positive affect and significantly reduce the negative affect of the subjects.

### Hypothesis 4 (H4)

The LKM intervention can significantly reduce the level of knowledge hiding of the subjects.

## Methods

### Participants

The subjects of this study were employees working in a knowledge-based enterprise in China. We published recruitment information through the internal network of the knowledge-based enterprise. Ultimately, there were 100 subjects who were eligible to participate and complete the experiment. The subjects were randomly divided into two groups: the LKM training group (50 participants) and the waiting control group (50 participants). There was no significant difference in demographic factors such as age composition and gender ratio composition between the two groups (Table 1).

**Table 1:** Demographic characteristics of participants

Characteristic	Total	LKM group	Control group
Age (SD)	38.83 (7.25)	38.62 (6.79)	39.04 (7.74)
Male (%)	62 (62%)	32 (64%)	30 (60%)
Female (%)	38 (38%)	18(36%)	20 (40%)

No demographic characteristic was significantly different among the two groups

### The instrument

#### Primary outcome measures

The Self-Report Altruism Scale (SRAS): This scale was developed by Rushton et al. in 1981. It is a self-report scale with 20 items, covering altruistic behaviors that often occur in people's daily life. The scale uses five-point Likert scores, from “1” meaning “never” to “5” meaning “very often”. It is a commonly used measurement tool for studying altruistic behavior [44]. The Chinese version of the SRAS, adapted by Chou, has the same structure [45]. In the current study, Cronbach’s alpha was 0.91.

The Knowledge Hiding Scale (KHS): This scale was developed by Connelly et al. and includes the three dimensions of Playing Dumb (PD), Rationalized Hiding (RH), Evasive Hiding (EH), with a total of 12 items. A seven-point Likert scoring method was used to answer the questions, ranging from totally disagree to fully agree [4]. The Chinese version of the KHS, adapted by Zhai, had the same structure [46]. In the current study, Cronbach's alpha for each dimension was: 0.87 for PD, 0.92 for RH, 0.88 for EH.

#### Secondary outcome measures

The Mindful Attention Awareness Scale (MAAS): This scale is used to measure individual characteristics of mindfulness and was developed by Brown and Ryan [47]. The Chinese version of the MAAS was adapted by Deng et al.; it has the same 15-item single-factor structure and a six-point Likert scale, where scores of 1 to 6 represent “almost always” to “almost never”. Higher scores indicate higher levels of mindfulness [48]. Among the many tools for measuring mindfulness traits, the MAAS is the most widely used. A large number of studies have shown that the MAAS has good reliability and validity in people with different cultural backgrounds [49]. In the current study, Cronbach’s alpha was 0.92.

The Positive and Negative Affect Scale (PANAS): This scale was developed by Watson, Clark and Tellegen, and was used to measure participants' positive affect [50]. The PANAS contains two dimensions: positive emotional experience and negative emotional experience. Each dimension has 10 items for a total of 20 items. Participants responded using a five-point Likert scale, where 1 meant “not at all” and 5 meant “all the time.” Previous studies have shown that the PANAS has good validity [51]. The Chinese version of the PANAS, adapted by Sheldon et al., has the same two-factor structure [52]. In the current study, Cronbach's alpha for each subdimension was: 0.88 for PA and 0.89 for NA.

### LKM intervention

The LKM intervention required 90 min per session five times a week, and lasted for a total of 8 weeks. Each 90-min session consisted of three consecutive phases: (1) up to 15 min of psychological education and warm-up topics, (2) 30 min of LKM practice and (3) a final discussion period of 45 min in which participants could share their LKM experiences with other participants and lecturers. For the LKM intervention, participants were asked to: (1) put themselves in a comfortable state, sometimes adding a pleasurable stimulus; (2) imagine the object to be blessed with compassion; (3) bless the object in four aspects: (a) wish him/her no enemies, (b) wish him/her no pain, (c) wish him/her no disease and (d) wish that he/she may have their own happiness. Regarding the selection of the object of blessing, the objects generally vary according to the level of the meditator's use of kindness meditation and follow the principle of going from easy to difficult. The general order is as follows: (a) bless oneself; (b) bless the people you love; (c) bless those who are neutral, that is, those who you neither like nor dislike; (d) bless the people you hate; (e) simultaneously and equally bless oneself, loved ones, neutral ones and hated ones; (f) bless all or all beings. When the practitioner is adept at arousing compassion for one type of person, the practice can move to a more difficult type of person. In addition, it is important to avoid some subjects, especially for beginners, during the practice of compassion meditation. For example, the object of blessing cannot be the opposite sex, cannot be too close to oneself or have the same interests, cannot be a dead person, and so on, in order to ensure the purity of the feelings cultivated [53].

### Procedure and design

The study included a two-arm randomized controlled trial in which the intervention condition was LKM practice and the control condition was waiting. The study used a 2(group) × 2(time) parallel trial design. Baseline measurements were taken immediately after randomization. The protocol for this study was reported according to the CONSORT guidelines. No changes have been made to methods after trial commencement, but the analytical methods have been modified on the recommendation of the reviewers.

**Fig. 1:**
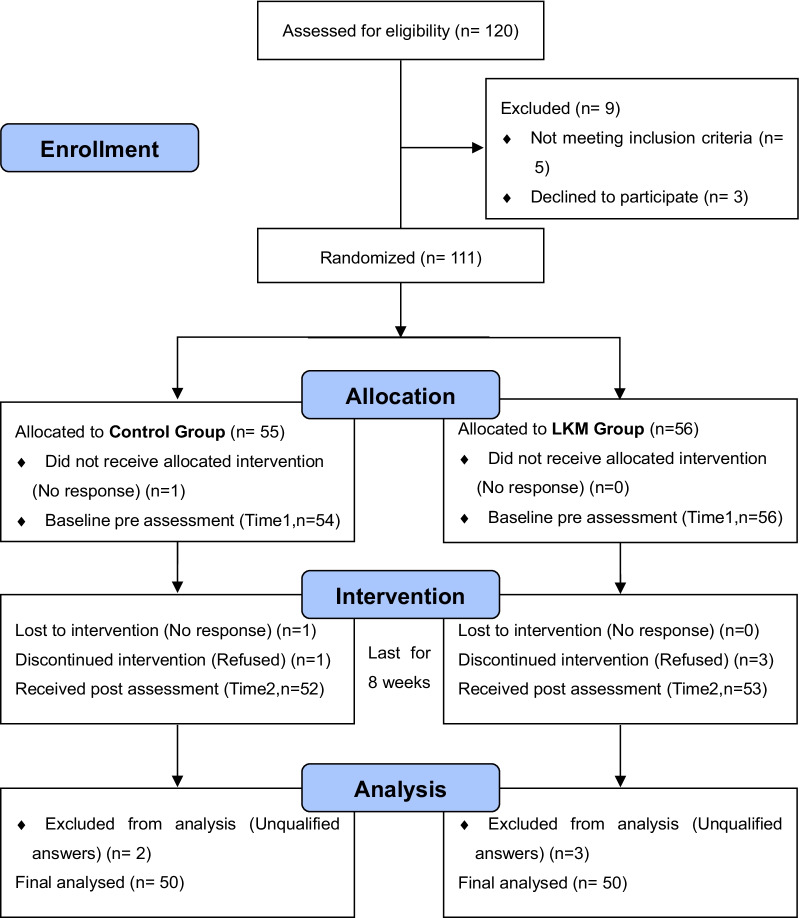
Procedure flow chart

We posted a recruitment advertisement for LKM on the internal network of a company in China, stating that LKM is a self-exploration activity to help employees better understand themselves and solve current problems. Employees who were interested and eligible to participate in our LKM study provided their registration information. Sample size was determined with G*Power. With 2 groups and 4 measurements, considering f = 0.3 at α = 0.05 and 80% of power, as well as a medium effect size of correlation among repeated measures. The recommended sample size for ANOVA with repeated measures was 95, and we expected an attrition rate of 20% from baseline to post-test, so we tried to recruit at least 120 participants. After screening, our baseline sample included 110 participants, of whom 100 qualified participants made it to the post-test. Figure 1 depicts the flowchart of experimental procedure and the participants through the study.

The grouping staff uses the random sequence code generated by SPASS 22 software for group assignment. These code lists, initially labeled “Group 1” and “Group 2,” were provided to participants, who were randomly assigned to either group 1 or group 2 using a 1:1 allocation ratio. The experimental intervention staff did not get a grouping list during the experimental intervention. After data analysis was completed, the grouping staff informed the data analysis staff and the experimental intervention staff of the meaning of each group in the group list. Through this procedure, double-blinding was achieved.

Inclusion criteria were: (1) bachelor degree or above and more than one year of working experience; (2) aged between 20 and 50; (3) agree to participate in this study and sign informed consent; (4) clear mind, normal thinking, can carry out language communication; (5) have sufficient cognitive ability and Chinese reading and writing ability to complete the questionnaire. Exclusion criteria were: (1) have a history of mental illness or mental disorders; (2) taking psychotropic drugs; (3) work without a permanent employer or freelance; (4) those who have engaged in any form of psychological intervention in the last year or currently; (5) have previous meditation training experience in any form. We recruited 120 subjects at first, and ultimately, there were 100 subjects who were eligible to participate and complete the experiment. We randomly assigned the 100 eligible participants to the LKM group (the LKM intervention group) or the control group (the waiting group). The 50 participants in the LKM group performed LKM meditation exercises together in a quiet, undisturbed rooms. The entire LKM intervention process was guided by a professional LKM teacher with 10 years of LKM practice experience and 2 years of meditation teaching experience, assisted by two researchers on site. The teacher was solely responsible for guiding the LKM exercises, and did not know the purpose of the study or participate in any other aspects of the study. The two researchers were solely responsible for on-site equipment debugging, handling emergencies, and recording and observing activity, and did not intervene in the teacher's guidance or participate in the training of the subjects.

At baseline test, each participant was told they were taking part in a study related to personality, and each participant received 50 RMB to boost their motivation. The participants were then provided with instructions, which the researchers made sure they understood before proceeding. After confirming that they understood the information, participants provided demographic data and completed the following questionnaires (pre-test): the Mindful Attention Awareness Scale (MAAS), the Self-Report Altruism Scale (SRAS), the Positive and Negative Affect Scale (PANAS) and the Knowledge Hiding Scale (KHS). It took about 30 min to answer the questionnaire. In addition, participants were asked about their overall satisfaction with the intervention to assess its acceptability. The subject has the right to terminate his or her participation in the study at any time without any reason, without prejudice to his or her legitimate rights and legal rights. The study facilitator may also suspend the study if necessary.

The duration of the LKM intervention was 8 weeks. At the end of the intervention period, participants completed the same questionnaire again (post-test) and received another 50 RMB as compensation. Finally, the real purpose of the study was explained to the participants. The study was approved by the Ethics Committee of Huaqiao University (IRB No.: 20200321) and the study protocol was carefully reviewed to ensure compliance with the ethical guidelines of the Chinese Psychological Society.

### Data analysis

The data was analyzed by SPSS 22. The confidence interval was set at 95%, and the significance level was set to 0.05. Descriptive statistics were used to describe the distribution of subjects' basic data, and ANOVA was used to compare the differences pre and post intervention.

## Results

**Fig. 2:**
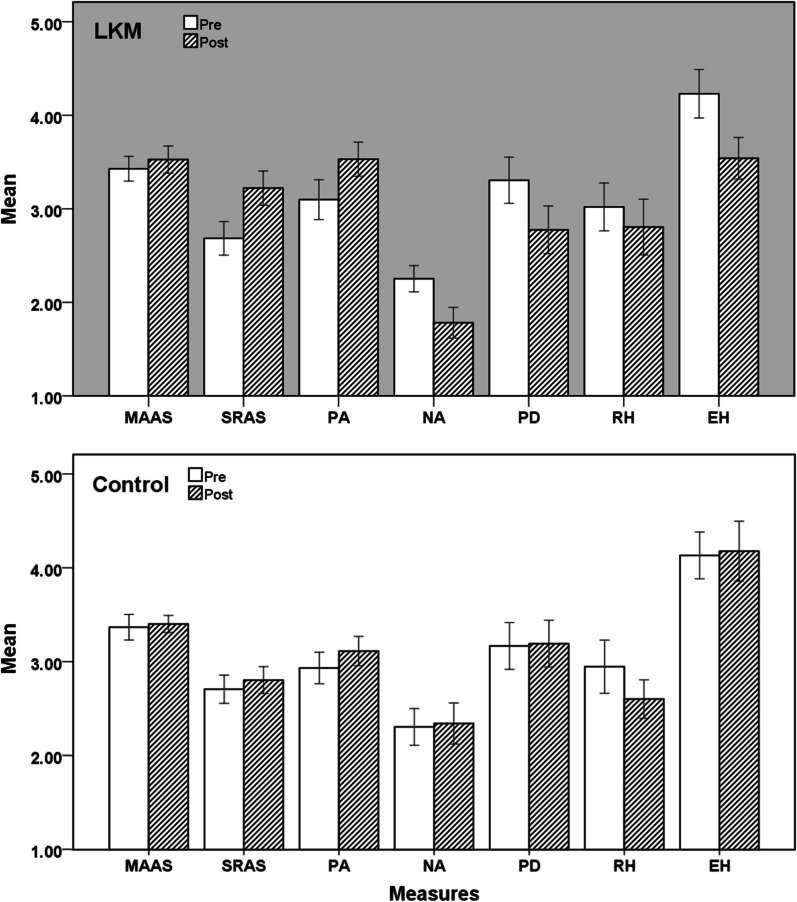
Pairwise comparison between LKM and control group. *Note.* *p < 0.05; ***p < 0.001. Only significant differences are marked with *, and those without * indicate that there is no significant difference; Errors bars: 95% Confidence Interval

Seven 2 (group type: LKM, control) × 2 (time: pre-test, post-test) ANOVAs with repeated measures (Fig. 2) were conducted on mindfulness (MAAS), altruism (SRAS), positive affect (PA), negative affect (NA) and the three dimensions of knowledge hiding (PD, RH and EH). For each group, number of participants included in each analysis and the analysis was by original assigned groups. The descriptive statistics are presented in Table 2, and the ANOVA results are presented in Table 3.

1. For mindfulness, there was no significant main effects of time or group, and no significant interaction between time and group. The results suggested that the LKM intervention did not significantly improve participants' mindfulness, which was not consistent with the hypothesis 1.
2. For altruism, there were significant main effects of time and group, and a significant interaction between time and group. As can be seen from the results, the LKM intervention significantly increased participants' level of altruism, which was consistent with the hypothesis 2.
3. For PA, there were significant main effects of time and group, but no significant interaction between time and group. The main effect of time without an interaction with group was not sufficient to confirm the hypotheses. For NA, there were significant main effects of time and group, and a significant interaction between time and group. These results revealed that the LKM intervention significantly reduced the NA level of the subjects, which was consistent with the hypothesis.
4. The three subdimensions of knowledge hiding were measured separately: for PD, there was a significant main effect of time but no significant main effect of group, and a significant interaction between time and group; for RH, only the main effect of time was significant; for EH, the main effects of time and group were significant and the interaction between them was significant. The results indicated the LKM intervention significantly reduced the two manifestations of knowledge hiding: playing dumb and evasive hiding, but had no significant effect on rationalized hiding. Hypothesis 4 was partially supported.

**Table 2:** Descriptive statistics

Group	Measures		Mean (SD)	
			Pre	Post
LKM	MAAS		3.428 (0.466)	3.526 (0.512)
	SRAS		2.684 (0.634)	3.221 (0.645)
	PA		3.098 (0.752)	3.530 (0.642)
	NA		2.252 (0.595)	1.782 (0.577)
	KHS	PD	3.305 (0.568)	2.775 (0.597)
		RH	3.020 (0.507)	2.805 (0.504)
		EH	4.230 (0.513)	3.540 (0.583)
Control	MAAS		3.370 (0.478)	3.404 (0.419)
	SRAS		2.709 (0.527)	2.806 (0.503)
	PA		2.936 (0.592)	3.116 (0.549)
	NA		2.308 (0.688)	2.344 (0.773)
	KHS	PD	3.170 (0.677)	3.195 (0.675)
		RH	2.950 (0.598)	2.605 (0.621)
		EH	4.135 (0.639)	4.180 (0.662)

**Table 3:** Results of ANOVA with repeated measures

Measure	Variable	F	*p*	η^2^
MAAS	Time	1.263	0.264	0.013
	Group	1.754	0.188	0.018
	Time × Group	0.295	0.588	0.003
SRAS	Time***	15.916	< 0.001	0.140
	Group*	5.302	0.023	0.051
	Time × Group**	7.666	0.007	0.073
PA	Time***	14.435	< 0.001	0.128
	Group**	8.465	0.004	0.080
	Time × Group	2.448	0.121	0.024
NA	Time*	6.265	0.014	0.060
	Group**	10.636	0.002	0.098
	Time × Group**	8.516	0.004	0.080
*KHS*				
PD	Time*	4.561	0.035	0.044
	Group	1.198	0.276	0.012
	Time × Group*	5.509	0.021	0.053
RH	Time*	4.591	0.035	0.045
	Group	1.060	0.306	0.011
	Time × Group	0.247	0.620	0.003
EH	Time*	5.406	0.022	0.052
	Group*	4.785	0.031	0.047
	Time × Group**	7.020	0.009	0.067

*p < 0.05; **p < 0.01; ***p < 0.001

The results of the above tables were integrated and presented in Fig. 2

In order to make the results more rigorous, Bonferroni’s method was used to adjust the *p*-values and confidence intervals, and the adjusted results are shown in Table 4. Pairwise comparisons were made only for variables with significant main effects.

**Table 4:** Adjustment with Bonferroni method for pairwise comparisons of main effects

Measure	Variable	Pairwise comparisons			
		Mean difference (Std. error)	*p*^a^	95% confidence interval^a^	
				Lower bound	Upper bound
SRAS	Time_(Pre, Post)_	− 0.317*** (0.079)	< 0.001	− 0.475	− 0.159
	Group_(L, C)_	0.195* (0.085)	0.023	0.027	0.363
PA	Time_(Pre, Post)_	− 0.306*** (0.081)	< 0.001	− 0.466	− 0.146
	Group_(L, C)_	0.288** (0.099)	0.004	0.092	0.484
NA	Time_(Pre, Post)_	0.217* (0.087)	0.014	0.045	0.389
	Group_(L, C)_	− 0.309** (0.095)	0.002	− 0.497	− 0.121
*KHS*					
PD	Time_(Pre, Post)_	0.253* (0.118)	0.035	0.018	0.487
	Group_(L, C)_	− 0.143 (0.130)	0.276	− 0.401	0.116
RH	Time_(Pre, Post)_	0.280* (0.131)	0.035	0.021	0.539
	Group_(L, C)_	0.135 (0.131)	0.306	− 0.125	0.395
EH	Time_(Pre, Post)_	0.323* (0.139)	0.022	0.047	0.598
	Group_(L, C)_	− 0.273* (0.125)	0.031	− 0.520	− 0.025

L. LKM; *C* control

*p < 0.05; **p < 0.01; ***p < 0.001

^a^Adjustment for multiple comparisons: Bonferroni

In order to further clarify the interaction effect of time and group, post-hoc analysis was conducted in this study, and the results are shown in Table 5. Bonferroni’s method was used to adjust the *p*-values and confidence intervals. Analyses were made only for variables with significant interaction effects.

**Table 5:** Adjustment with Bonferroni method for pairwise comparisons of interaction effects

Measure	Time × Group	Pairwise comparisons			
		Mean difference (Std. error)	*p*^a^	95% confidence interval^a^	
				Lower bound	Upper bound
SRAS	Pre_(L, C)_	− 0.025 (0.117)	0.831	− 0.256	0.206
	Post_(L, C)_	0.415*** (0.116)	< 0.001	0.185	0.645
	L_(Pre, Post)_	− 0.537*** (0.112)	< 0.001	− 0.760	− 0.314
	C_(Pre, Post)_	− 0.097 (0.112)	0.390	− 0.320	0.126
NA	Pre_(L, C)_	− 0.056 (0.120)	0.641	− 0.294	0.182
	Post_(L, C)_	− 0.562*** (0.136)	< 0.001	− 0.833	− 0.291
	L_(Pre, Post)_	0.470*** (0.123)	< 0.001	0.227	0.713
	C_(Pre, Post)_	− 0.036 (0.123)	0.770	− 0.279	0.207
*KHS*					
PD	Pre_(L, C)_	0.135 (0.174)	0.441	− 0.211	0.481
	Post_(L, C)_	− 0.420* (0.177)	0.020	− 0.772	− 0.068
	L_(Pre, Post)_	0.530** (0.167)	0.002	0.198	0.862
	C_(Pre, Post)_	− 0.025 (0.167)	0.881	− 0.357	0.307
EH	Pre_(L, C)_	0.095 (0.179)	0.597	− 0.260	0.450
	Post_(L, C)_	− 0.640** (0.194)	0.001	− 1.024	− 0.256
	L _(Pre, Post)_	0.690*** (0.196)	< 0.001	0.301	1.079
	C_(Pre, Post)_	− 0.045 (0.196)	0.819	− 0.434	

L. LKM; *C* control

*p < 0.05; **p < 0.01; ***p < 0.001

^a^Adjustment for multiple comparisons: Bonferroni

Our hypotheses stated that LKM should significantly increase mindfulness, altruism and positive affect, and significantly reduce negative affect and knowledge hiding. The results showed that H2 was supported, H3 and H4 were partially supported, and H1 was not supported.

## Discussion

### LKM intervention

LKM intervention significantly reduced negative affect. First, since LKM has the function of purifying negative affect. Another expression used for the concept of purifying in positive psychology is breaking through the original mental dysfunction to achieve spiritual improvement. There are many obstacles in human psychological function. If people cannot break through the obstacles, they will experience many psychological problems in their consciousness, such as selfishness, greed, anger, hostility and depression [54]. Most of these psychological problems are maladaptive habits and reaction patterns caused by past experience. When people experience something unpleasant, they instinctively tend to try to prevent the experience from happening again, but in the long run, this avoidance strategy often makes things worse by reinforcing their habit of responding to problems [55]. People always unconsciously form various hypotheses and inferences based on their initial experience, and constantly seek biased evidence to reinforce them, thus forming wrong ideas and rigid thinking habits, so negative affect and behaviors may occur during this process [56]. LKM can redirect these experiences in a warm way, channeling negative affect and perhaps even replacing these negative thoughts with compassion [57]. Secondly, because LKM plays a role in developing positive affect, brain imaging studies have shown that the reward circuit is significantly activated in LKM meditators as they process other people's misfortune cues [58]. Studies using videos with different levels of emotional arousal as experimental stimuli, found that when watching high-arousal videos of other people suffering misfortune, both novice and experienced compassion meditators who had been trained in LKM compared with control groups showed significantly enhanced activation of brain regions such as the ventral striatum, the orbital region of the MFC and ventral tegmental area, and self-reported positive affect were significantly increased [59, 60]. These brain regions are important parts of the brain's reward network and are associated with the social connections individuals experience, as well as positive affect and motivations associated with prosocial orientations, such as love, kindness and compassion for others [61, 62]. Although the LKM intervention in this study did not significantly improve the positive affect of the subjects, it significantly reduced their negative affect. The reason may be that all the subjects were exposed to LKM training for the first time and did not have skilled meditation techniques and rich meditation experience, so LKM did not have a particularly strong impact on their emotional state and only reduced their negative affect. This point will be further explored and verified in the future research.

LKM intervention significantly improved participants' altruistic behavior. First, LKM enhanced the subjects' empathic response to the miseries of others by cultivating empathy. When a person sees someone in need, he/she is likely to feel empathy for the other person [63]. Altruistic people are more likely to empathize with others and put themselves in others' shoes when motivated by external motives. [64]. Most psychologists believe that all altruistic behavior can eventually produce self-rewarding results. Why do altruists help others without expecting anything in return? Batson proposed an “empathy–altruism” hypothesis to explain this phenomenon. He argued that when we see someone in need, the first thing that influences our decision is whether we feel empathy for that person. Do we empathize when this person appears distressed and helpless? If we have empathy, we will help him/her regardless of the cost [65]. According to this hypothesis, altruistic help occurs when people feel a strong sense of empathy for those in need [66]. Second, LKM significantly reduced the subjects’ negative affect and promoted their positive affect to some extent. Previous studies on emotions and interpersonal interaction have found that the social function of positive affect can make individuals more willing to help others and more willing to help colleagues and establish more social resources. George and Brief proposed that there is a correlation between positive emotion and helping behavior. Positive emotion may prime individual memory, making individuals more positive towards others and more positive in assessing opportunities to help others. Therefore, individuals in a positive emotional state are more likely to show helping behavior [67]. Mood is also a factor in helping behavior. Research shows that people are more motivated to do good deeds when they are in a good mood [68]. Some scholars believe there are three reasons for this. First, a happy mood makes people pay more attention to the bright side of life, pay more attention to the strengths of others and see people in a positive light, so they are more motivated to help others [69]. Second, doing good deeds can prolong a good mood, forming a virtuous circle [70]. Third, a good mood can increase people’s self-attention and make people more likely to present themselves according to their ideal image. When faced with the situation of someone seeking help, the individual’s mood at that time will affect the helping behavior. Positive affect promotes altruistic behavior, and individuals in positive states of mind, including happiness experienced in response to success, love and pleasant surprises, are more willing to help [71]. A study evoked positive affect by offering participants a windfall of unexpected money (a coin). When the lucky person picked up the coin, he or she was confronted by a lab assistant holding a folder that had been "accidentally" dropped a few meters away from the subject. Altruistic behavior was judged on whether or not the subject stepped forward to help pick up the folder. The results showed that 4% of individuals in the control group who did not receive a windfall helped the lab assistant pick up the folder, while 84% of those who had just received a coin acted altruistically [72].

LKM intervention significantly reduced the knowledge hiding behavior in two ways: playing dumb and evasive hiding. Firstly, LKM serves as a means to regulate emotions when someone is in bad mood. Positive affect can make employees broaden their resources and opportunities, making it easier for them to recover their energy to cope with future challenges. Negative affect refers to the negative feelings of exhaustion and boredom experienced by employees [73]. Such types of negative affect are not conducive to the acquisition of resources by employees, and it takes a while for employees to recover their energy. Therefore, they do not have enough energy to help others when they are asked for help [74]. If the request is complex and the requester has a poor understanding of the question, it will consume a lot of energy and time to reply to the requester's question, so that staff in a resource-exhausted state will tend to hide their knowledge. The social function of emotion can reduce knowledge hiding among team members [75]. Secondly, the emergence of individual knowledge hiding behavior originates from individual spontaneous decisions, so it will be affected by individual states or characteristics. It is found that under positive affect, individuals are more confident and optimistic, more direct when making requests, more willing to cooperate with others, less confrontational when sharing opinions, more willing to help others and displaying fewer knowledge hiding behaviors [76]. The sharing and acceptance of information and views will affect the collision of thinking between individuals and others, and individuals experiencing positive affect will show more helpful behaviors [77]. During negative affect, individuals are more likely to criticize others' opinions and give negative feedback, and are more willing to hide knowledge [78]. Thirdly, LKM improved the prosocial motivation of the practitioner. Motivation dominates human’s goal-directed behavior [79]. Many studies have found that LKM training enhanced the practitioner’s prosocial motivations, including empathy for the victim and affiliation [80]. The reason may be because in the process of meditation, the practitioner's blessing of the imaginary object enhances the perceived social connection and stimulates the practitioner's prosocial motivation, which directly reduces knowledge hiding. Some studies have found that LKM training can significantly enhance the functional connections of the dorsolateral prefrontal cortex and nucleus accumbens. Enhancement of this circuit could positively predict the helpful behavior of individuals in a game [81]. The nucleus accumbens not only plays a key role in reward processing, but also receives fiber afferences from prefrontal cortex and other regions, and is deeply involved in an individual’s behavior selection and the process of approaching motivation-related stimuli [82]. These findings suggest that the reward network of compassion meditators is more active in the face of other people's suffering, which can improve the expectation of helping others to get out of pain and gaining inner warmth and satisfaction, stimulating the prosocial motivation of meditators and reducing knowledge hiding.

In this study, LKM intervention had no significant effect on rationalized hiding. The reason may be that the rationalization of the way of hiding can make the way appear more legitimate by means of a grand reason, more easily accepted by the requestor. For the consideration of business competition, knowledge-based enterprises usually pay more attention to the protection of intellectual property rights [83]. Enterprises and employees usually sign various confidentiality and non-competition agreements to ensure that employees' knowledge leakage behaviors will not cause harm to the enterprise during their employment and for a period of time after leaving the company. These institutional frameworks also limit the dissemination of knowledge to a certain extent and provide a reasonable way to hide knowledge [84]. Secondly, LKM can also help to improve subjects' sense of moral responsibility and make them abide by rules and regulations more. Organizational norms are patterns of organizational behavior, attitudes, and beliefs. These models are established by social organizations in formal or informal ways and are considered appropriate codes of conduct [85]. In countries that value collectivism, organization members are more inclined to value collective interests over individual interests, and attach more importance to rigor and the protection of corporate resources [86]. In addition, when subjects adopt the strategy of pretending to be stupid or evading concealment, those with high moral responsibility may feel guilty for deceiving others, and are likely to think that knowledge concealment is caused by their own reasons, that is, internal motivational attribution [87]. However, when subjects use rationalized hiding strategy, because the subjects provided cannot disclose knowledge justifiable reasons (e.g., confidentiality agreement), the subjects are likely to think that the emergence of hidden knowledge is caused due to some external system or others, to produce the external motivation attribution, because the subjects considered hidden knowledge out of duty, thus reducing their sense of guilt and moral responsibility [88]. These reasons may result in the limited impact of LKM on rationalized hiding.

In this study, LKM intervention did not significantly increase participants' mindfulness. Mindfulness is the process of paying attention to what is happening in the present moment—including internal experiences (thoughts, bodily sensations) and external stimuli (physical and social environments), and observing these stimuli without judging, reacting to, or giving them meaning [89]. Although both mindfulness and LKM emphasize high concentration of attention, mindfulness requires maintaining awareness of internal experience and external stimuli while focusing attention, while LKM subjects are completely immersed in the process of blessing others [90]. As LKM practitioners are highly focused on compassionate blessings, their external awareness is greatly reduced and their awareness of external stimuli is affected, making it difficult for them to achieve a state of mindfulness. In addition, the non-judgmental response mode of mindfulness to all stimuli also conflicts with the immersive blessing state of LKM [91]. Previous studies have found that immersion is negatively correlated with mindfulness awareness, which may partly explain why LKM exercises fail to significantly improve mindfulness. A high degree of immersion is an important attribute of flow state [92]. Flow is a state of mind in which people engage in certain behaviors. It is the feeling of being fully involved in an activity [93–95]. Flow experiences are associated with high levels of excitement and satisfaction. In this study, LKM did not significantly improve participants' mindfulness level, but it may stimulate their flow, which needs to be further verified in future studies.

## Research limitations and future studies

This study has the following limitations. First, the sample size was relatively small due to limitations in the number of employees, scientific research funds and the nature of the work of employees from the same company. Second, due to the occupational nature and busy work of employees, it was impossible to place them in a completely closed experimental environment for 8 weeks of intervention. Beyond this 8-week LKM intervention, subjects had to continue to participate in daily work and life. Any interference the subjects encountered during the intervention was not within the control of the study. Fourth, the four scales used in this study are all self-report scales, with a certain degree of egocentric error. Therefore, the current results should be thought of as perceived mindfulness, positive affect, altruism and knowledge hiding. Fifth, this study only used one LKM model, and different models may produce different results. Sixth, Chinese society is mainly influenced by Confucian culture. Apart from blood relationships, trust between people is relatively low. Therefore, our study may have cross-cultural problems. It is suggested to look at the effect of LKM on knowledge hiding in different countries in the future. Lastly, the study design lacked an active control group controlling for effects of the LKM group that were not specific to LKM practices. Hence, it cannot be ruled out that some of the benefits observed for the LKM group maybe trace back to demand characteristics (e.g., psychoeducational input) or positive interactions with the LKM teacher or the intervention group, not only to LKM practices in the narrower sense.

Since the broaden-and-build theory of positive affect was put forward, more and more studies have confirmed that positive affect is an important asset in the workplace because the variables relate to positive mood and many important work outcomes (such as creativity, customer service, quality, support for organizational change, etc.) [11]. Therefore, future organizational psychology researchers may make full use of this theory to conduct in-depth research on positive organizational behavior.

First, the relationship between positive affect, and employee and organizational growth can be studied in the future. By experiencing positive affect, employees can become more creative, knowledgeable, resilient, socially engaged and healthy. These positive results are exactly what the organization’s managers are happy to see. Moreover, the broaden-and-build theory of positive affect indicates that creating a self-sustaining system in an upward spiral allows for sustained employee growth. Emotion is contagious and can be socially contagious between organization members and between organization members and customers. Thus, positive affect has adaptive significance for individual employees, and as the upward spiral of positive affect continues, positive affect can make organizations run well and help them thrive and prosper. Therefore, in-depth research on the positive significance of positive affect for employees and organizations is worth further exploration.

Second, future studies could consider how to manage negativity in organizations. Negativity in the workplace is inevitable, and negative affect is also adaptive. Therefore, it may be necessary to maintain a reasonable ratio of positive to negative affect in an organization. A study showed that a high positive-to-negative affect ratio in organizations was correlated with high team performance [96]. At present, although it is not clear where the tipping point for positive emotion is for an organization, it is worth exploring further.

## Conclusions

The current study provides a detailed and nuanced understanding of the effects of LKM on mindfulness, altruism, affect, and knowledge hiding. The results showed that LKM significantly improved employees’ altruism, and significantly reduce their negative affect, but did not significantly improve their mindfulness and positive affect. For knowledge hiding, LKM significantly reduced playing dumb and evasive hiding, but had no significant effect on rationalized hiding. These results further elucidate the psychological effects of LKM and suggest the possibility of reducing knowledge hiding in the workplace. The mechanism of LKM’s influence on mindfulness, positive and negative affect, altruism, knowledge hiding and other psychological constructs needs to be clarified further.

## Data Availability

The datasets during the current study are not publicly available due to privacy restrictions but are available from the first author on reasonable request.

## Abbreviations

EH: Evasive hiding
KHS: Knowledge hiding scale
LKM: Loving-kindness meditation
MAAS: Mindful attention awareness scale
NA: Negative affect
PA: Positive affect
PANAS: Positive and negative affect scale
PD: Playing dumb
RH: Rationalized hiding
SRAS: Self-report altruism scale
